# Population Pharmacokinetic Modelling of Remdesivir and Its Metabolite GS-441524 in Hospitalised Patients with COVID-19

**DOI:** 10.1007/s40262-025-01496-2

**Published:** 2025-04-22

**Authors:** Darren M. Roberts, Xin Liu, Suzanne L. Parker, Andrew Burke, Jenny Peek, Jane E. Carland, Bridin Murnion, Vincent Seah, Steven C. Wallis, Chandra D. Sumi, Saurabh Pandey, Hergen Buscher, Anthony Byrne, Indy Sandaradura, David Bowen, Simon Holz, Adam G. Stewart, Krispin M. Hajkowicz, Jason A. Roberts

**Affiliations:** 1 The University of Queensland Centre for Clinical Research, Faculty of Health, Medicine and Behavioural Sciences, The University of Queensland, Brisbane, Australia; 2 Department of Clinical Pharmacology and Toxicology, St Vincent’s Hospital Sydney, Darlinghurst, Australia; 3 School of Clinical Medicine, Faculty of Medicine and Health, St Vincent’s Healthcare Clinical Campus, University of New South Wales, Sydney, Australia; 4 Drug Health Services, Edith Collins Centre, Royal Prince Alfred Hospital, Sydney, Australia; 5 Department of Infectious Diseases, The Prince Charles Hospital, Brisbane, Australia; 6 Department of Intensive Care Medicine, St Vincent’s Hospital Sydney, Sydney, Australia; 7 Thoracic Medicine, St Vincent’s Hospital Sydney, Sydney, Australia; 8 Centre for Infectious Diseases and Clinical Microbiology, Westmead Hospital, Sydney, Australia; 9 Faculty of Medicine and Health, Westmead Clinical School, The University of Sydney, Sydney, Australia; 10 Institute of Clinical Pathology and Medical Research, New South Wales Health Pathology, Westmead Hospital, Sydney, Australia; 11 Department of Intensive Care Medicine, Westmead Hospital, Sydney, Australia; 12 Herston Infectious Diseases Institute (Heidi), Metro North Health, Brisbane, Australia; 13 Department of Infectious Diseases, Royal Brisbane and Women’s Hospital, Brisbane, Australia; 14 Departments of Pharmacy and Intensive Care Medicine, Royal Brisbane and Women’s Hospital, Brisbane, Australia; 15 Division of Anaesthesiology Critical Care Emergency and Pain Medicine, Nîmes University Hospital, University of Montpellier, Nîmes, France

## Abstract

**Background and Objectives:**

There are limited data testing whether the licensed dose of remdesivir and its active metabolite GS-441524 achieve target concentrations in hospitalised patients with confirmed severe acute respiratory syndrome coronavirus-2 (SARS-CoV-2), the cause of coronavirus disease-2019 (COVID-19). The objectives of this study were to describe the population pharmacokinetics of remdesivir and GS-441524 in hospitalised patients treated for COVID-19 and develop a model to inform dose optimisation in clinical use.

**Methods:**

This was a prospective, open-labelled, multi-centre, observational study in four Australian hospitals in adults with confirmed SARS-CoV-2 infection. Patients were administered the licensed remdesivir dose. Remdesivir and GS-441524 concentrations were quantified in multiple plasma samples at different times in the dosing interval by ultra-high-performance liquid chromatography-mass spectrometry/mass spectrometry (LC-MS/MS). Patients were divided into two groups: pharmacokinetic model building and external validation. A population pharmacokinetic analysis was built using non-linear mixed-effects modelling. Monte Carlo simulations were performed to describe the impact of age, kidney function and dosing regimen on drug concentrations.

**Results:**

In total, 33 patients were enrolled (median age 70 years, estimated glomerular filtration rate (eGFR) 80 mL/min/1.73 m^2^). The pharmacokinetics for both compounds were adequately described by a two-compartment model (one compartment for each compound) with first-order elimination. Key covariates included in the final model were age and eGFR. GS-441524 plasma concentrations exceeded the lowest reported half-maximal effective concentration (EC_50_) with the recommended dosage, and higher dosages exceeded the lowest reported 90%-effective concentration (EC_90_).

**Conclusions:**

The licensed remdesivir dose may achieve target concentrations of GS-441524, but higher dosages may optimise outcomes. Dose adjustments are guided primarily by kidney function.

**Supplementary Information:**

The online version contains supplementary material available at 10.1007/s40262-025-01496-2.

## Key Points

**Table:** 

An integrated population pharmacokinetic model was developed and externally validated for both remdesivir and GS-441524 in patients hospitalized with COVID-19.
The concentration of GS-441524 was substantially higher in patients with reduced kidney function due to decreased clearance.
The concentration of GS-441524 was substantially higher in patients with older age due to a smaller volume of distribution.
This model can guide dosing of remdesivir in patients of varying ages and health.

## Introduction

Remdesivir (formerly GS-5734) is a monophosphoramidate nucleoside analogue drug that was originally developed as a treatment for Ebola virus in response to the 2014–2016 outbreak in West Africa [1]. Remdesivir is administered intravenously and then bioconverted to an intermediate alanine metabolite, GS-704277, and then nucleotide monophosphate metabolite, GS-441524, in plasma [2]. The GS-441524 metabolite enters cells and is then converted to the pharmacologically active triphosphate analogue, GS-443902. Remdesivir and its metabolites show antiviral activity against severe acute respiratory syndrome coronavirus-2 (SARS-CoV-2), the cause of coronavirus disease-2019 (COVID-19), inhibiting the viral RNA-dependent RNA polymerase [3]. Remdesivir therapy retains activity against Delta, Omicron variants and its subvariants, with varying potency described [4–11].

Intravenous remdesivir is approved or provisionally approved for the treatment of COVID-19 in adult and paediatric patients in many countries. The approved intravenous dosage was based on data in healthy volunteers and pharmacokinetic modelling, being a 200 mg loading dose followed by 100 mg once daily for 5 days [12]. There is little data to confirm whether licensed remdesivir dosing regimens achieve effective exposures (concentrations) in patients with COVID-19 of varying age, weight and/or organ function.

The main aim of the current study was to describe the plasma concentrations of GS-441524 with the recommended dosing regimen in hospitalised patients treated for COVID-19 and to use the available data to develop a population pharmacokinetic model of both remdesivir and GS-441524. We further aimed to use the final model to define optimised dosing regimens for hospitalised patients treated for COVID-19.

## Patients and Methods

### Patients and Study Design

This study was a prospective, open-labelled, multi-centre, observational study performed at four Australian hospitals between July 2021 and August 2022. Patients over 18 years old admitted to hospital with confirmed SARS-CoV-2 infection (nucleic acid amplification test positive within 14 days of symptom onset) were eligible for inclusion if they were prescribed remdesivir for treatment of COVID-19. Informed consent was obtained from all participants, or their responsible person, prior to enrolment. Data on patient demographics (age, sex, weight, height, body mass index (BMI) and ethnicity), clinical characteristics (comorbidities, smoking status, oxygenation, ventilation status, medications, antibiotic use, antiviral use, immunomodulatory agents and sepsis organ function assessment (SOFA) score) and clinical laboratory investigations (aspartate aminotransferase (AST), alanine aminotransferase (ALT), alkaline phosphatase (ALP), gamma-glutamyl transferase (GGT), platelet count, white cell count, albumin, and bilirubin and serum creatinine concentrations) were collected from the patient’s medical record. Estimated glomerular filtration rate (eGFR) was calculated on the basis of serum creatinine concentrations using the chronic kidney disease epidemiology collaboration formula (CKD-EPI) formula [13], with appropriate caution being applied in patients with significant acute kidney injury [14]. Patients with eGFR < 30 mL/min/1.73 m^2^ were excluded from this pharmacokinetic study.

Remdesivir was administered as an intravenous infusion over 60 min at a loading dose of 200 mg on day 1 followed from day 2 up to day 5 or day 10 as a 100 mg dose once daily over 60 min. Blood samples were collected in sodium fluoride/potassium oxalate anticoagulant tubes (for measuring remdesivir and GS-441524 concentrations) or potassium EDTA (if measuring GS-441524 concentrations only). Where possible, multiple samples were collected over one dosing interval on two separate occasions. Occasion 1 occurred on day 1–3 of remdesivir therapy and occasion 2 occurred on a different day between days 3–7 of therapy. Samples were collected at pre-dose, 1.5, 3, 6, 8–12 and 20–24 h after commencement of infusion. Surplus plasma from samples obtained for clinical care at other times during the dosing interval were also obtained, where possible.

Samples for measuring remdesivir and GS-441524 were placed in an ice water bath immediately on collection owing to the instability of remdesivir at room temperature [15, 16]. Samples were centrifuged within 1 h of collection and 1–2 mL plasma were transferred to polypropylene cryovial for storage at –80 °C for later drug analysis. Surplus plasma samples for measuring GS-441524 (GS-441524 is stable at room temperature for 48 h [17]) were stored refrigerated in the pathology laboratory until being processed within 48 h of collection.

### UHPLC-MS/MS Assay

Plasma concentrations of remdesivir and GS-441524 were determined by an ultra-high performance liquid chromatography coupled with tandem mass spectrometry (UHPLC-MS/MS) method. Chromatographic separation was achieved with a Kinetex C8, 100 mm × 2.1 mm (1.7 μm) analytical column (Phenomenex, Torrence, USA) preceded by C8 UHPLC guard cartridge (Phenomenex, Torrence, USA). Mobile phase A was 0.1% formic acid in water (*v*/*v*), and mobile phase B was 0.1% formic acid in acetonitrile (v/v). Mobile phase was delivered as a gradient from 30 to 90% of mobile phase B over 6 min at a flow 0.30 mL/min. Remdesivir and GS-441524 were monitored by positive-mode electrospray at multiple reaction monitoring (MRM) of m/z 603.20→229.05 and 291.95→163.15, respectively. The lower limit of quantification (LLOQ) was 5 ng/mL for both remdesivir and GS-441524. Test samples were assayed in batches alongside calibrators and quality controls and the results were subject to pre-established batch acceptance criteria [18].

### Population Pharmacokinetic Analysis

Patients contributing plasma concentrations were divided into model-building and external validation datasets at an approximate ratio of 3:1, prioritising patients with complete data collection to the model-building cohort. The non-linear mixed-effects modelling program Monolix version 2021R2 (Lixoft SAS, a Simulations Plus company, Antony, France), implementing the stochastic approximation expectation maximization (SAEM) algorithm, was used. An integrated model containing both remdesivir and its metabolite GS-441524 was developed. To account for the differences in molecular weight (MW) between remdesivir (MW = 602.585) and GS-441524 (MW = 291.26), concentration data were converted from ng/mL to µM.

One-, two- and three-compartment models with first order elimination were tested as base structural models for remdesivir and GS-441524. As 10% of the administered remdesivir is excreted unchanged in the urine [19], renal clearance of remdesivir was fixed to 10% of the total remdesivir clearance. All individual parameters were assumed to be log-normally distributed. The between-subject variability (BSV or *ω*) was described using an exponential model. To describe the residual variability (*ε*), several error models (constant, proportional or combined error model) were tested. The most appropriate model was selected on the basis of the following criteria: the objective function value (OFV) (defined as –2 × log-likelihood, –2LL), usual goodness-of-fit (GOF) plots, and relative standard error (RSE) of parameter estimates.

### Covariate Analysis

From the base model, the effects of the following covariates on remdesivir and GS-441524 pharmacokinetic parameters, with consideration of biological plausibility, were evaluated: age, gender, height, body weight, BMI, eGFR, SOFA score, serum albumin, bilirubin, ALT, AST, ALP and GGT level. Continuous covariates were modelled using linear and power functions.

The covariate model was built using a stepwise procedure with forward inclusion and backward elimination. The statistical significance of each covariate was individually evaluated during the stepwise deletion using the likelihood ratio test (LRT). The covariate was retained in the model if the effect was biologically plausible, it produced a reduction in BSV of the parameter and the OFV was decreased by at least 3.84 (*P* < 0.05) when included (as compared with the base model) and increased by 6.63 (*P* < 0.01) when eliminated (as compared with the model with the covariate included).

### Model Evaluation

Evaluation of the model was based on goodness-of-fit (GOF) plots, including observations versus individual and population predictions and plots of normalized prediction distribution error (NPDE) versus population predictions and time. A visual predictive check (VPC) was performed using 500 simulations with the final model. This plot shows the time course of the 5th, 50th and 95th percentiles of the simulated profiles, compared with observed data.

The accuracy of the final model was also examined using a bootstrap method. A 1000-run bootstrap resampling procedure was performed in Monolix using the Rsmlx (R Speaks ‘Monolix’, version 4.0.2) package in R software (version 4.1.3). The median, 2.5% and 97.5% values obtained from the 1000 bootstrap runs for each parameter were calculated and compared with the estimates from the original data.

### External Validation

GS-441524 concentrations from a sub-set of patients were used for external validation to assess the predictive performance of the final model. The relative prediction error (PE, Eq. 1) of the model was estimated by comparing the population predicted concentrations (*C*_pred_) and the corresponding observations (*C*_obs_) for each subject at each time point in the external validation dataset. Mean prediction error (MPE) and the root mean square error (RMSE, Eq. 2) were calculated to assess the prediction bias and precision. Bland–Altman plots were developed to visualize the trends of bias. The population pharmacokinetic model was regarded as valid when both the mean and median values of PE were less than 20% [20].

1 $$PE = \left(\frac{{C}_{\text{pred}} -{ C}_{\text{obs}}}{{C}_{\text{obs}}}\right)\times 100,$$

2 $$RMSE=\sqrt{\frac{1}{N}\sum {(PE)}^{2}}.$$

### Dosing Simulations

Monte Carlo simulations (*n* = 1000) were performed by Simulx version 2021R2 (Lixoft SAS, a Simulations Plus company, Antony, France) using the final pharmacokinetic model to generate pharmacokinetic profiles of remdesivir and GS-441524 for a 5-day remdesivir treatment regimen. A typical virtual patient with COVID-19 with age of 70 years and an eGFR of 80 mL/min/1.73 m^2^ was used to simulate the pharmacokinetic profiles of remdesivir and GS-441524 for six 5-day remdesivir dosing regimens: (1) currently licensed dosage of 200 mg loading dose followed by 100 mg once daily; (2) a loading dose of 200 mg followed by 150 mg once daily; (3) a 200 mg loading dose on day 1 followed by 100 mg every 12 h; (4) a loading dose of 100 mg followed by 50 mg every 6 h for 5 days in total; (5) a loading dose of 300 mg followed by 50 mg every 6 h for 5 days in total as recommended by others[21]; and (6) 200 mg dose on day 1 and day 2. All doses were simulated as a 1 h intravenous infusion and were chosen to explore highly varied dosing regimens which may inform alternative and potentially more effective dosing regimens. To investigate the effect of the eGFR and age on GS-441524 pharmacokinetics, simulations were performed on the basis of different eGFRs (40, 80, 120 mL/min/1.73 m^2^) and ages (30, 70 and 90 years). The standard licensed dosing regimen was used for these simulations.

As the clinical pharmacodynamics of remdesivir have not been described, we used two target exposures, an unbound plasma concentration above the in vitro half-maximal effective concentration (EC_50_) and above the 90%-effective concentration﻿ (EC_90_) for remdesivir and GS-441524. Literature-reported EC_50_ or EC_90_ values for remdesivir and GS-441524 are summarised in the Supplementary Material Table S2. These values were corrected for protein binding, 88% for remdesivir and 2% for GS-441524, to compare directly with measured plasma concentrations of remdesivir or GS-441524 [22].

### Ethics Approval

The study protocol was approved by Human Research Ethics Committees of St Vincent’s Hospital Sydney (2021/ETH11567), The University of Queensland (2022/HE00260) and Royal Brisbane and Women’s Hospital (HREC/2020/QRBW/66533).

## Results

### Characteristics of Patients

**Fig. 1:**
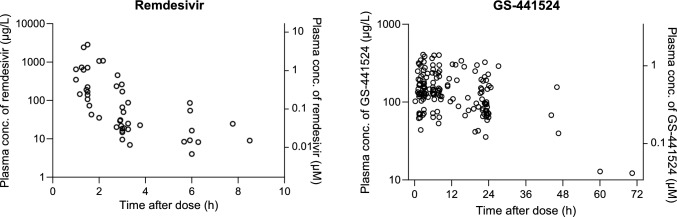
Plasma concentration-time profiles of remdesivir and GS-4411524 after intravenous infusion of remdesivir in the studied population. Concentrations are expressed as µg/L in left *y*-axis and as µM in right *y*-axis

The median age of the 33 patients recruited to this study was 70 years (interquartile range (IQR) 60–77 years, range 25–97 years). The median baseline eGFR of the population was 80 mL/min/1.73 m^2^ with a range of 33–124 mL/min/1.73 m^2^. In addition, 21 patients (64%) were male. No patient had preexisting chronic liver disease nor received kidney replacement or vasopressor therapy. Overall, 72% of patients were prescribed oxygen therapy, 30% were admitted to the intensive care unit, and 12% were prescribed ventilatory support, including one patient requiring endotracheal intubation. The baseline and demographic characteristics for both the model development and external validation groups are presented in Table 1. Plasma concentration-time profiles of remdesivir and GS-4411524 are shown in Fig. 1.

**Table 1:** Characteristics of patients included in this study

Characteristics	Model building data (*n* = 25)	External validation data (*n* = 8)
**Demographics**		
Gender ratio, M/F	15/10	6/2
Age (years)	69 (IQR 60–73; range 25–97)	78 (IQR 64–80; range 25–86)
Weight (kg)	92 (IQR 65–104; range 44–132)	Not recorded
Body mass index	30 (IQR 26–33; range 17–46; *n* = 24)	23 (*n* = 1)
**Baseline clinical characteristics**		
Cardiovascular disease	10 (40%)	4 (50%)
Lung disease, e.g. chronic obstructive pulmonary disease (*n* = 2 current smokers)	4 (16%)	1 (13%)
Prescribed immunosuppressants	4 (16%)	1 (13%)
Diabetes mellitus	5 (20%)	2 (25%)
Chronic kidney disease (eGFR 30–60 mL/min/1.73 m^2^)	10 (40%)	1 (13%)
Serum creatinine concentration (µmol/L)	88 (IQR 72–110; range 40–195)	81 (IQR 72–88; range 64–127)
eGFR (mL/min/1.73 m^2^)	72 (IQR 59–87; range 34–124)	82 (IQR 79–90; range 33–108)
AST (U/L)	29 (IQR 24–42; range 19–109)	30 (IQR 21–39; range 6–43; *n* = 6)
ALT (U/L)	22 (IQR 17–42; range 5–102)	19 (IQR 13–35; range 8–51)
Albumin (g/L)	31 (IQR 27–35; range 22–39; *n* = 24)	34 (IQR 30–35; range 29–36)
**Clinical management of COVID-19**		
Prescribed oxygen therapy	16 (64%)	8 (100%)
Admitted to intensive care unit	7 (28%)	3 (38%)
Prescribed mechanical ventilatory support	4 (16%)^$^	0 (0%)
Co-prescribed immunomodulatory drugs		
Corticosteroids, mostly dexamethasone	22 (88%)	8 (100%)
Baricitinib	12 (48%)	8 (100%)
Tocilizumab	2 (8%)	0 (0%)
Sotrovimab	2 (8%)	0 (0%)
Co-prescribed antibiotics	6 (24%)	1 (13%)
**Overall outcomes**		
Survived to discharge from hospital	24 (96%)	8 (100%)
Acute kidney injury	1 (4%); stage 1*	1 (13%); stage 1*
Worst disease severity in survivors		
Mild	5 (20%)	3 (38%)
Moderate	4 (16%)	3 (38%)
Severe	10 (40%)	2 (25%)
Critical illness	5 (20%)	0 (0%)

Data are expressed as median (interquartile range; range)

ALT, alanine aminotransferase; AST, aspartate aminotransferase; eGFR, estimated glomerular filtration rate; IQR, interquartile range

*Acute kidney injury graded according to the KDIGO classification [29], on the basis of an increase in serum creatinine above baseline, where stage 1 is 1.5–1.9 times baseline or > 26.5 µmol/L

^$^One patient was mechanically ventilated

### Population Pharmacokinetic Model

In total, 49 remdesivir plasma concentrations from 12 patients and 153 GS-441524 concentrations from 25 patients were used for model development. The observed plasma concentration-time profiles of remdesivir and GS-441524 are presented in the Supplementary Material (Supplementary Fig. S1). A two-compartment model with first-order elimination was selected as the best structural model to describe the pharmacokinetics of both remdesivir and GS-441524 (Supplementary Fig. S2). BSV was estimated for the volume of distribution and clearance of both remdesivir and GS-441524. The combined (proportional plus additive) and proportional error model were used to describe residual variability for remdesivir and GS-441524, respectively.

In the covariate analysis, adding eGFR on clearance of GS-441524 using a power model improved the model fit, decreased the OFV by 22 points and decreased the BSV of clearance by 22%. Adding age on volume of distribution (V) of GS-441524 using a power model further improved the model fit, decreased OFV by 8 points and explained 12% of the BSV of *V*. Backward elimination of these covariates significantly increased OFV over 6.84 (*p* < 0.01) (Supplementary Table S1). The following equations were used to define the individual eGFR-scaled clearance and the age-scaled *V* for GS-441524.

$$\text{GS-}441524\,{ \left(\frac{Cl}{{f}_{\text{m}}}\right)}_{\text{i}}=15.9\times {\left(\frac{{eGFR}_{\text{i}}}{80}\right)}^{1.12},$$

$$\text{GS-}441524\,{ \left(\frac{V}{{f}_{\text{m}}}\right)}_{\text{i}}=429\times {\left(\frac{{Age}_{i}}{68.5}\right)}^{-1.15},$$

where (*Cl/f*_m_*)*_*i*_ is apparent clearance estimate for the *i*th individual, *eGFR*_*i*_ is the eGFR value for the *i*th individual, (*V/f*_m_)_*i*_ is the apparent volume of distribution estimate for the *i*th individual and *Age*_i_ is the age for the *i*th individual.

The final estimates of population pharmacokinetic parameters for remdesivir and GS-441524 are summarised in Table 2.

**Table 2:** Estimates of population pharmacokinetic parameters of the final model

Parameter	Final model			Bootstrap	
	Mean	RSE (%)	Shrinkage (%)	Median	95% CI
**Remdesivir**					
CL (L/h)	105	17.8		100	69.3–154
*V* (L)	121	23.7		111	45.9–195.5
**GS-441524**					
CL/*f*_m_ (L/h)	15.9	8.39		16.0	13.2–19.6
*V*/*f*_m_ (L)	429	11.4		429	353–512
Age effect on V	–1.15	33.7		–1.18	– 2.82 to – 0.51
eGFR effect on CL	1.12	0.02		1.23	0.78–1.89
**Between subject variability (%)**					
Remdesivir CL	53.1	29.5	10.0	45.4	9.03–77.7
Remdesivir V	61.4	31.2	12.8	60.2	14.6–122
GS-441524 CL/*f*_m_	36.8	17.7	6.91	34.4	20.1–50.1
GS-441524 *V*/*f*_m_	46.0	22.1	–5.52	40.4	18.9–67.5
**Error model parameter**					
Remdesivir additive residual (µM)	0.014	38.1		0.016	0.0008–0.039
Remdesivir proportional error	0.62	17.2		0.61	0.44–0.74
GS-441524 proportional error	0.16	7.06		0.16	0.11–0.20

CL, clearance; CI, confidence interval;  *f*_m_, fraction of remdesivir metabolized to GS-441524; RSE, relative standard error; *V*, volume of distribution

**Fig. 2:**
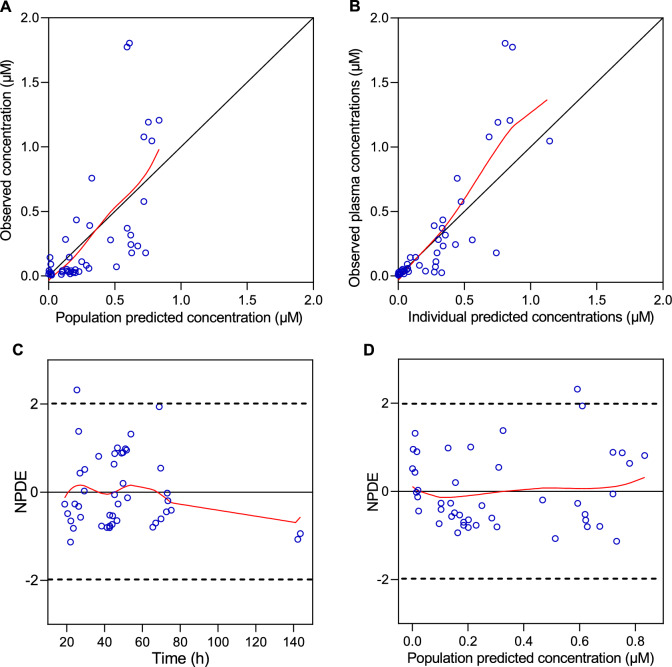
The GOF plots of remdesivir. **A** Observed versus population predicted concentrations; **B** observed versus individual predicted concentrations. The solid black line represents the line of identity, and the solid red line represents the spline line; **C** NPDE versus time; **D** NPDE versus population predicted concentrations. The solid red line represents the spline line

**Fig. 3:**
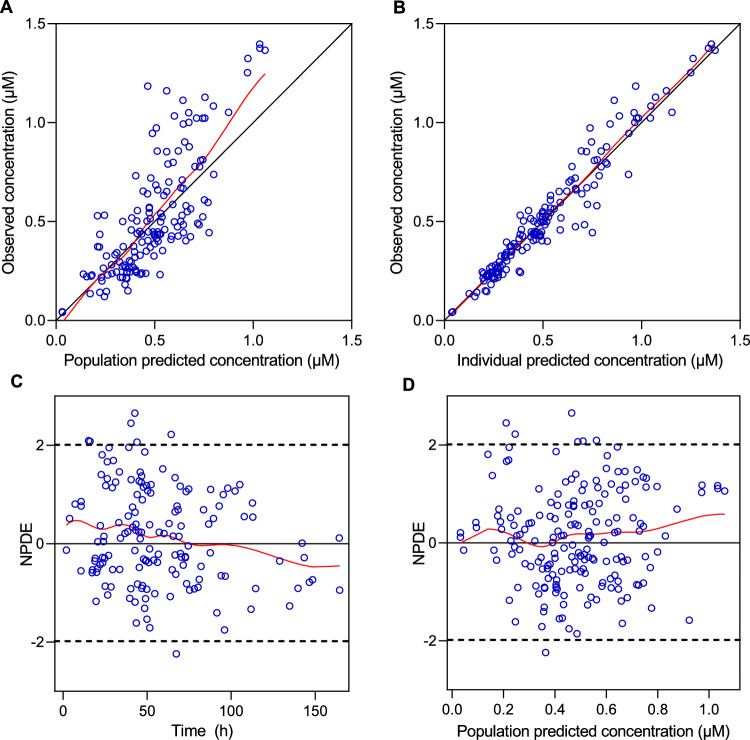
The GOF plots of GS-441524. **A** Observed versus population predicted concentrations; **B** observed versus individual predicted concentrations. The solid black line represents the line of identity, and the solid red line represents the spline line. **C** NPDE versus time; **D** NPDE versus population predicted concentrations. The solid red line represents the spline line

**Fig. 4:**
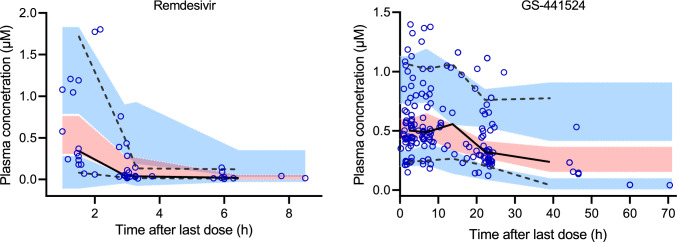
VPC plots. Dots represent observations. Lines represent empirical percentiles (5, 50 and 95 percentile). Shaded area represents 90% prediction interval around each predicted percentile (5, 50 and 95 percentile)

The median values of the non-parametric bootstrapping replicates were close to the final estimates of all parameters (Table 2), confirming the estimated values. The goodness-of-fit (GOF) plots (Figs. 2 and 3) indicated a good fit of the model to both remdesivir and GS-441524 in the targeted population. Visual predictive check (VPC) plots (Fig. 4) for plasma concentrations of remdesivir and GS-441524 versus time profiles showed that the model describes the population tendency and variability of the data.

The final model was valid based on the external validation with median and mean PE of –15.2% and –19.5%, respectively. The RMSE of 30.5% indicates a reasonable precision of the model prediction. The Bland–Altman plot (Supplementary Fig. S3) shows that there was no explicit trend in the PE over the whole concentration range observed in the external data set, although there might be a consistent but slight underestimation. It was noted that the median age of the external validation cohort was older than the model development cohort and this may have led to the slight underestimation of plasma concentrations in the external validation dataset using the typical population estimate of *V/fm* from the model development cohort.

### Monte Carlo Simulations

**Fig. 5:**
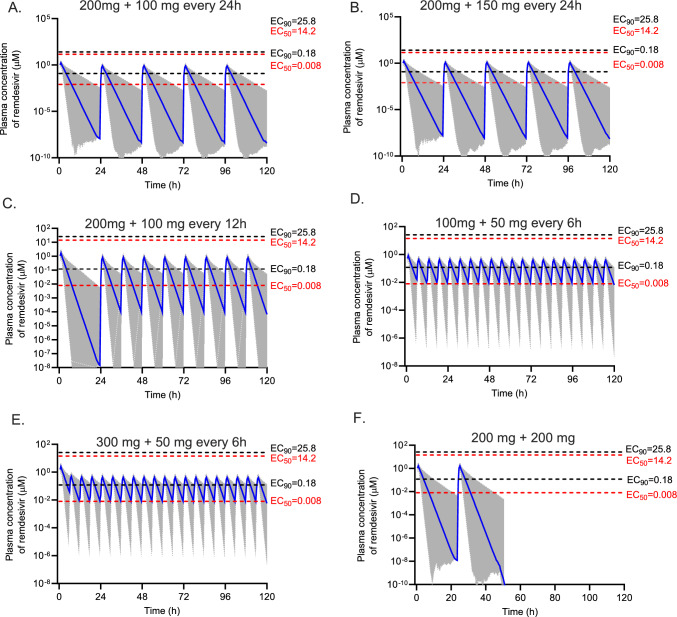
Simulated remdesivir concentrations versus time profiles for six dosing regimens. The blue line is the median concentration, and the shaded area is the 95% prediction interval. The red dash lines represent the lowest and highest EC_50_ of remdesivir against SARS-CoV-2 variants reported in the literature, corrected for plasma protein binding. Similarly, the black dash lines represent the lowest and highest EC_90_

**Fig. 6:**
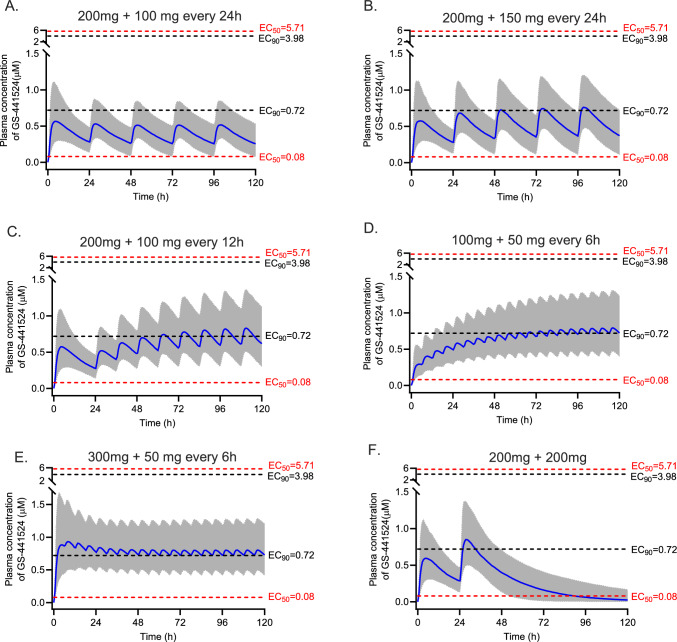
Simulated GS-441524 plasma concentrations versus time profiles for six dosing regimens. The blue line is the median concentration, and the shaded area is the 95% prediction interval. The red dash lines represent the lowest and highest EC_50_ of GS-441524 against SARS-CoV-2 variants reported in the literature, corrected for plasma protein binding. Similarly, the black dash lines represent the lowest and highest EC_90_

Simulated plasma concentration-time profiles of remdesivir and GS-441524, Figs. 5 and 6, respectively, describe five alternative dosing regimens for patients with eGFR values at the median level of 80 mL/min/1.73 m^2^ and age of 70 years. The simulated median trough concentrations of GS-441524 ranged from 0.26 µM (standard dosing regimen) to 0.72 µM (maintenance dose 50 mg every 6 h, achieved 48–72 h sooner with 300 mg loading dose, compared with 100 mg). Increasing the frequency of remdesivir dosing leads to an increase in the plasma concentrations of GS-441524, Fig. 6.

**Fig. 7:**
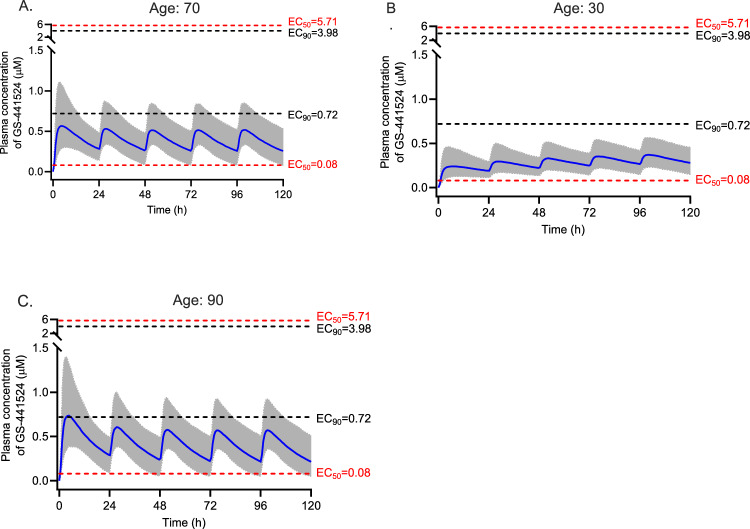
Simulated GS-441524 plasma concentrations versus time profiles for three age groups with eGFR of 80 mL/min/1.73 m^2^ using the standard dosing regimen of 200 mg followed by 100 mg every 24 h. The blue solid line is the median concentration, and the shaded area is the 95% prediction interval. The red dash lines represent the lowest and highest EC_50_s against SARS-CoV-2 variants corrected for plasma protein binding. The black dash lines represent the lowest and highest EC_90_

**Fig. 8:**
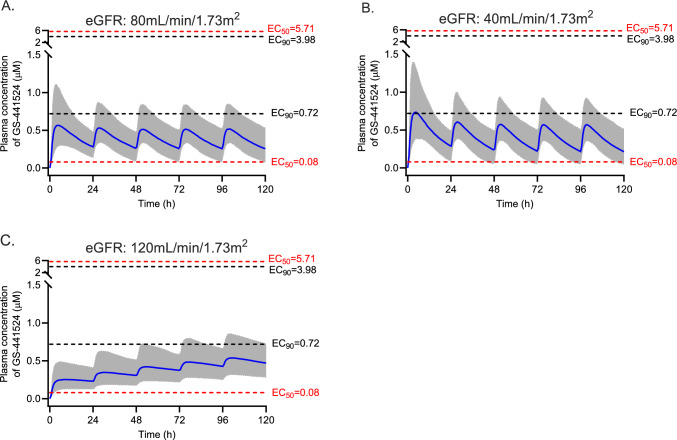
Simulated GS-441524 plasma concentrations versus time profiles for three different eGFR with age of 70 years using the standard dosing regimen of 200 mg followed by 100 mg every 24 h. The blue solid line is the median concentration, and the shaded area is the 95% prediction interval. The red dash lines represent the lowest and highest EC_50_s against various SARS-CoV-2 variants corrected for plasma protein binding. The black dash lines represent the lowest and highest EC_90_

The influence of age and the eGFR on GS-441524 pharmacokinetics are shown in Figs. 7 and 8, respectively. Older patients show higher plasma concentrations compared with younger patients as evidenced by median trough concentration of 0.28 µM versus 0.22 µM. Compared with eGFR 80 mL/min/1.73 m^2^, the plasma concentrations of GS-441524 were lower for patients with an eGFR of 120 mL/min/1.73 m^2^ and higher for patients with a reduced eGFR (< 40 mL/min/1.73 m^2^). The simulated median trough concentrations of GS-441524 were 0.22, 0.26 and 0.47 µM for eGFR of 120, 80 and 40 mL/min/1.73 m^2^, respectively.

The simulated plasma concentrations of remdesivir and GS-44152 are compared with plasma protein binding corrected EC_50_ and EC_90_ values reported in literature in Supplementary Table S2.

## Discussion

This study is the first to develop an integrated population pharmacokinetic model that was externally validated for both remdesivir and GS-441524 in patients hospitalized with COVID-19. The pharmacokinetics for both compounds were adequately described by a two-compartment model (one compartment for each compound). The pharmacokinetic profiles of GS-441524 were affected significantly by both eGFR and age, whereby concentrations were substantially higher in those with reduced eGFR (due to decreased clearance) and older age (due to smaller volume of distribution). This was further substantiated with Monte Carlo simulations using the final model and a range of dosages. Monte Carlo dosing simulations indicated that decreasing the remdesivir dosing interval would lead to an increase in GS-441524 plasma concentrations which may optimise the effect of remdesivir by achieving the EC_90_.

A wide range of EC_50_ (0.001–1.7 µM for remdesivir and 0.08–5.6 µM for GS-441524) and EC_90_ (0.022–3.1 µM for remdesivir and 0.71–3.9 µM for GS-441524) values are reported in the literature for remdesivir and GS-441524, depending on the variants of virus, cell type and in vitro assay methods used. Therefore, evaluating drug efficacy on the basis of these in vitro values will give different conclusions. To obtain reliable predictions of the probability of target attainment, more studies are required to determine the in vivo target range of plasma concentrations. This is emphasised by the outcomes of this study that found that for the standard dosing regimen in different patient populations, along with other dosing regimens explored, GS-441524 concentrations were frequently below the lowest EC_90_ reported. This increases the likelihood that these patients are at risk of treatment failure. On the basis of simulation results, the dosing interval of remdesivir should be reduced to achieve higher trough GS-441524 concentrations, ensuring optimal treatment outcomes. These simulations can also be used to define dosing regimens that should be used for new EC_50_ and/or EC_90_ data from future SARS-CoV2 variants or other target concentrations identified in the future for this or other viruses.

Population pharmacokinetics of remdesivir and GS-441524 have been described in 17 hospitalized patients with COVID-19 (84 blood samples) in the Netherlands by Leegwater et al. [22]. The median age of the population was 55 years (range 31–74 years) and the median eGFR was 94 mL/min/1.73 m^2^ (range 8–119 mL/min/1.73 m^2^). A population pharmacokinetic model of GS-441524 alone was also developed in two Japanese COVID-19 patient cohorts [23, 24]. The median age of the first cohort was 72 years (range, 45–97 years) and the median eGFR was 74.7 mL/min (range 16.4–147.7) [23]. The second cohort had a median age of 70 years (range 42–85) and median eGFR of 68 mL/min (range 33–113). As in our study, a two-compartment model was used to describe pharmacokinetics of both remdesivir and GS-441524, with one compartment for each compound.

Remdesivir clearance (96.2 L/h) and volume of distribution (90.3 L) obtained in our study was lower than that reported for Dutch patients with COVID-19 (207 L/h and 157 L, respectively [22]). GS-441524 clearance (15.9 L/h) and volume of distribution (429 L) found in our study were comparable to those in Japanese patients (11.8 L/h and 382 L [23] and 11.0 L/h and 271 L [24]) but were lower than those in Dutch patients [22] (27.6 L/h and 1060 L, respectively). Across all studies, these parameters in patients with COVID-19, as well as healthy volunteers [25], were affected by the eGFR and age. The higher clearance and volume of distribution of GS-441524 observed in the Dutch patients might be due to the higher eGFR and lower age in that population compared with our study and the Japanese patient group. Conversely, simulations also show the much higher GS-441524 concentrations in patients with COVID-19 with severely decreased kidney function (Fig. 7). Sukeishi et al. recommended dose reductions on the basis of eGFR owing to this observation [23]. However, Leegwater et al. [22] argued against this recommendation because several observational studies indicated no increased adverse effects in patients with eGFR less than 30 mL/min/1.73 m^2^. More recently the REDPINE study described outcomes in 249 patients with impaired kidney function enrolled in a placebo-controlled trial in which the intervention group received the licensed dose of remdesivir [26]. Practically, this resulted in these patients receiving a much higher exposure of GS-441524 compared with patients with normal kidney function prescribed the same dose. Although underpowered owing to slow recruitment, adverse events were similar between both groups, but clinical benefits were not observed. The high incidence of adverse effects in both arms, including an overall mortality of 30% in randomised patients, may complicate the risk–benefit assessment of this dosing regimen in this patient population. Our work largely supports these findings, including possible benefits from higher dosages (e.g. Fig. 6E) and that eGFR impacts GS-441524 plasma concentrations, which can inform the remdesivir dosing regimen to achieve target concentrations of GS-441524 for COVID-19 or other susceptible viruses identified in the future.

In this study, we identified the significant effect of eGFR on GS-441524 clearance, which was consistent with previous reports [22–24]. The major limitation of the current study is the limited sample size and sample number per dosing interval. However, we separated the dataset into model-developing and external validation datasets, which allows us to provide a robust validation of the model, providing confidence in its value and utility. Furthermore, our simulations were for remdesivir total plasma concentrations. We compared those concentrations with the corrected EC_50_s and EC_90_s on the basis of remdesivir having a protein binding of 88%, informed by previous studies [22] and experimental data (88–94%) [27]. However, a more recent study in patients infected with COVID-19 indicated that protein binding was 97% [28]. If protein binding is closer to 97% than 88% in clinical conditions then the corrected EC_50_s and EC_90_s in Figs. 5, 6, 7, 8 and Supplementary Table S2 would be higher, so the probability of target attainment would decrease. Finally, our predictions on the probability of target attainment focus on GS-441524 plasma concentrations because GS-441524 dominates the concentration-time profile (Figs. 1 and 4) and is a more potent inhibitor of viral replication (Supplementary Table S2). However, this approach does not consider the potential additional effect of intracellular penetration and accumulation of both remdesivir and GS-441524. For example, in vitro studies indicate that remdesivir readily penetrates cells and results in higher intracellular GS-441524 concentrations than would be achieved by administration of GS-441524 [10], which may support including remdesivir concentrations when evaluating the probability of target attainment. However, the intracellular partitioning of remdesivir and GS-441524 changes with time [27] and data describing temporal changes of these intracellular concentrations during the treatment course are limited. A clinical study noted that concentrations of the active triphosphate metabolite GS-443902 increased in peripheral blood mononuclear cells 2.04-fold with each additional infusion of remdesivir in non-pregnant people [28]. This relatively slow accumulation of intracellular concentrations also prompts consideration of alternative dosing regimens with higher loading doses, such as those explored in our study.

## Conclusions

This study presents the integrated population pharmacokinetic model for remdesivir and GS-441524 in hospitalized patients with COVID-19 without significant kidney disease. In these patients, remdesivir clearance was variable but comparable to that in heathy volunteers, and GS-441524 clearance was informed by eGFR.

## Supplementary Information

Below is the link to the electronic supplementary material.

Supplementary file1 (PDF 275 KB)

## Declarations

### Availability of Data and Material

The data that support the findings of this study are not openly available owing to reasons of sensitivity but are available from the corresponding author upon reasonable request.

### Code Availability

Modelling script is included in the Supplementary File and can be made available upon reasonable request.
