# Supplementary material for: Population Pharmacokinetic Modelling of Remdesivir and Its Metabolite GS-441524 in Hospitalised Patients with COVID-19
Source: Clin Pharmacokinet. 2025 Apr 22;64(5):743–56. doi: 10.1007/s40262-025-01496-2 (PMC12064607; doi:10.1007/s40262-025-01496-2)
Supplement: Supplementary file 1 — Supplementary file1 (PDF 275 KB) [file 40262_2025_1496_MOESM1_ESM.pdf]

Supplementary material

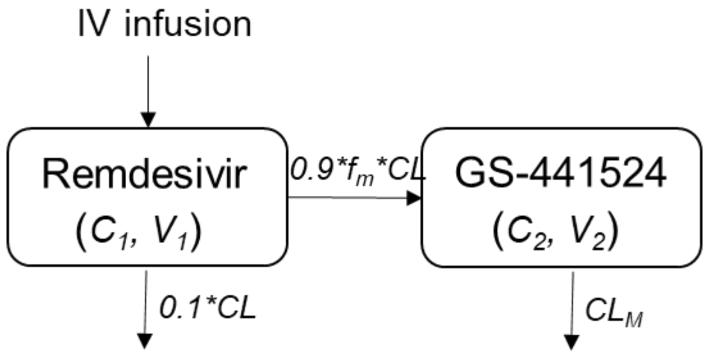

Fig S1. Schematic diagram of the final pharmacokinetic model for remdesivir and GS-441524. CL, total clearance of remdesivir; V_1, volume of distribution of remdesivir; f_m, the fraction of remdesivir metabolized to GS-441524; CL_M, clearance of GS-441524; V_2, volume of distribution of GS-441524.

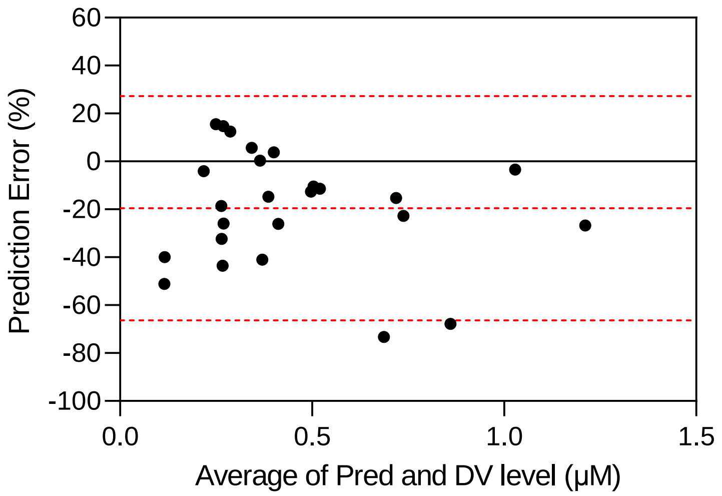

Fig S2. Bland-Altman plot based on the external data set showing the predictive performance of the final model.

Table S1. Summary of the model building processes during covariate analysis

Process	Model	OFV	Δ OFV	BSV_CL(%)	BSV_V(%)
Base model	2-compartment	-275.06	-	59	56
Stepwise forward addition	Add eGFR effect on CL_M	-296.72	-21.66	37	51
	Add eGFR effect on CL_M and age effect on V_2	-304.81	-29.76	36	44
Backward elimination	Remove eGFR effect on CL_M	-283.58	+21.23	54	47
	Remove age effect on V_2	-296.72	+8.1	37	51

Table S2. Summary of literature reported EC50s and EC90s of remdesivir and GS-441524 against various variants of SARS-CoV-2.

Variant	Cell line	Assay	Remdesivir				GS-441524				Ref.
			EC_{50} (μM)	[a]Corrected EC_{50} (μM)	EC_{90} (μM)	[a]Corrected EC_{90} (μM)	EC_{50} (μM)	[b]Corrected EC_{50} (μM)	EC_{90} (μM)	[a]Corrected EC_{90} (μM)	
2019-nCoV/USA-WA1/2020	Calu3 2B4	Plague	0.28	2.33	2.48	20.7	0.62	0.63	1.34	1.37	Pruijssers et al[9]
		qRT-qPCR	0.60	5.00	1.28	10.7	1.09	1.11	1.37	1.40	
	HAE	Plague	0.01	0.083							
	Vero E6	Plague	1.65	13.7	2.40	20.0	0.47	0.48	0.71	0.72	
		qRT-qPCR	1.49	12.4	3.03	25.3	0.47	0.48	0.80	0.82	
nCoV-2019BetaCoV/Wuhan/WIV04/2019	Vero E6	qRT-PCR	0.77	6.42	1.76	14.7					Wang et al[11]
BetaCoV/France/IDF0571/2020	Vero E6	RT-qPCR	0.99	8.25							Pizzorno et al[8]
BetaCoV/Belgium/GHB-03021/2020	Vero E6	$TCID_{50}$	0.74-1.34	6.17-11.2			0.78-0.89	0.80-0.91			Do et al[7]
	HAE		0.048	0.40			0.51	0.52			
USA-WA1/2020	Vero E6	qRT-PCR	1.0	8.33	3.1	25.8	1.1	1.12	3.9	3.98	Tao et al[10]
	Vero CCL-81		0.7	5.83	1.7	14.2	0.8	0.82	1.6	1.63	
	Calu-3		0.11	0.92	0.49	4.08	0.25	0.26	2.35	2.40	
	Caco-2		0.001	0.008	0.022	0.18	0.08	0.08	1.42	1.45	
WA1	A549-ACE2-TMPRSS	Nucleo-protein ELISA	0.11	0.92			5.6	5.71			Pitts et al[4]
Delta			0.07	0.58			3.26	3.33			

Omicron			0.042	0.35		3.15	3.21		
Omicron/BA.2	Vero E6	Focus reduction				2.85	2.91		Takashita et al[5]
UT-NC002-1T/Human/2020/Tokyo	Vero-hACE2-TMPRSS2	Focus reduction	1.7	14.2					Takashita et al[6]

[a] corrected for 88% plasma protein binding
[b] corrected for 2% plasma protein binding

MONOLIX model file

[LONGITUDINAL]

```
input = {V1,CL,V2,CLm,eGFR,beta}
eGFR={use=regressor}

Pharmacokinetic (PK):
depot(target= A1)

EQUATION:
Initial condition
t_0=0
A1_0=0
A2_0=0

CLr=0.1*CL                    ; renal clearance of remdesivir
k10=CLr/V1                    ; rate constant of renal elimination of remdesivir
CLtr=0.9*CL                   ; metabolism clearance of remdesivir
k12=CLtr/V1                   ; remdesivir transformation rate constant to GS-441524
CLmi=CLm*(eGFR/80)^beta ; covariate effects of eGFR on clearance of GS-441524
k20=CLmi/V2                   ; elimination rate constant of GS-441524

ddt_A1 = -(k10+k12)*A1        ; central compartment of remdesivir
ddt_A2 = k12*A1 - k20*A2      ; central compartment of GS-441524

C1 = A1/V1                    ; plasma concentration of remdesivir
C2 = A2/V2                    ; plasma concentration of GS-441524

OUTPUT:
output={C1, C2}
```

Table S3. Definition of disease severity for adults (https://app.magicapp.org/#/guideline/L4Q5An/section/nV2P3n)

**Mild illness**	An individual with no clinical features suggestive of moderate or more severe disease:<br><br>• no OR mild symptoms and signs (fever, cough, sore throat, malaise, headache, muscle pain, nausea, vomiting, diarrhoea, loss of taste and smell)<br>• no new shortness of breath or difficulty breathing on exertion<br>• no evidence of lower respiratory tract disease during clinical assessment or on imaging (if performed)
**Moderate illness**	A stable patient with evidence of lower respiratory tract disease:<br><br>• during clinical assessment, such as<br>○ oxygen saturation 92–94% on room air at rest<br>○ desaturation or breathlessness with mild exertion<br>• or on imaging
**Severe illness**	A patient with signs of moderate disease who is deteriorating<br><br>OR<br><br>A patient meeting any of the following criteria:<br><br>• respiratory rate ≥ 30 breaths/min<br>• oxygen saturation < 92% on room air at rest or requiring oxygen<br>• lung infiltrates > 50%
**Critical illness**	A patient meeting any of the following criteria:<br><br>• Respiratory failure (defined as any of)<br>○ severe respiratory failure ($PaO_2/FiO_2 < 200$)<br>○ respiratory distress or acute respiratory distress syndrome (ARDS)<br>○ deteriorating despite non-invasive forms of respiratory support (i.e. non-invasive ventilation [NIV], or high-flow nasal oxygen [HFNO])<br>○ requiring mechanical ventilation<br>• hypotension or shock<br>• impairment of consciousness<br>• other organ failure
